# Identification and Validation of a Prognostic Model Based on Three Autophagy-Related Genes in Hepatocellular Carcinoma

**DOI:** 10.1155/2021/5564040

**Published:** 2021-03-12

**Authors:** Fanbo Qin, Junyong Zhang, Jianping Gong, Wenfeng Zhang

**Affiliations:** ^1^Department of Hepatobiliary Surgery and Chongqing Key Laboratory of Hepatobiliary Surgery, The Second Affiliated Hospital of Chongqing Medical University, Chongqing 400010, China; ^2^Department of Urology Surgery, The Second Affiliated Hospital of Chongqing Medical University, Chongqing 400010, China

## Abstract

**Background:**

Accumulating studies have demonstrated that autophagy plays an important role in hepatocellular carcinoma (HCC). We aimed to construct a prognostic model based on autophagy-related genes (ARGs) to predict the survival of HCC patients.

**Methods:**

Differentially expressed ARGs were identified based on the expression data from The Cancer Genome Atlas and ARGs of the Human Autophagy Database. Univariate Cox regression analysis was used to identify the prognosis-related ARGs. Multivariate Cox regression analysis was performed to construct the prognostic model. Receiver operating characteristic (ROC), Kaplan-Meier curve, and multivariate Cox regression analyses were performed to test the prognostic value of the model. The prognostic value of the model was further confirmed by an independent data cohort obtained from the International Cancer Genome Consortium (ICGC) database.

**Results:**

A total of 34 prognosis-related ARGs were selected from 62 differentially expressed ARGs identified in HCC compared with noncancer tissues. After analysis, a novel prognostic model based on ARGs (PRKCD, BIRC5, and ATIC) was constructed. The risk score divided patients into high- or low-risk groups, which had significantly different survival rates. Multivariate Cox analysis indicated that the risk score was an independent risk factor for survival of HCC after adjusting for other conventional clinical parameters. ROC analysis showed that the predictive value of this model was better than that of other conventional clinical parameters. Moreover, the prognostic value of the model was further confirmed in an independent cohort from ICGC patients.

**Conclusion:**

The prognosis-related ARGs could provide new perspectives on HCC, and the model should be helpful for predicting the prognosis of HCC patients.

## 1. Introduction

Hepatocellular carcinoma (HCC) is the fifth most common cancer worldwide and the third most common cause of cancer mortality [1]. The main cause of HCC is nonalcoholic fatty liver disease (NAFLD) and hepatitis virus infection including chronic hepatitis B virus (HBV) infection and chronic hepatitis C virus (HCV) infection [2]. In America, although the 5-year survival rate of HCC patients increased by 90% between 2006 and 2012 compared to 1992-1999, the overall prognosis of HCC remains poor [3]. Therefore, establishing an effective prognostic model can provide new guidance for clinical management. However, conventional clinical parameters used to predict clinical outcome often have some limitations given the heterogeneity of HCC [4]. Therefore, the establishment of a novel effective prognostic model must be based on the molecular heterogeneity of HCC.

Autophagy is a lysosomal degradation pathway for unwanted, damaged, and defective intracellular components and is essential for survival, differentiation, development, and homeostasis [5]. In recent decades, accumulating evidence has demonstrated that abnormal expression of autophagy-related genes (ARGs) is involved in the development of various cancers [6, 7]. However, the role of ARGs in cancer remains inconclusive. In general, at the early stage of liver cancer, autophagy protects cells from carcinogenesis. At an advanced stage, however, autophagy can promote cancer progression [8, 9]. For liver cancer, dysregulated autophagy is dependent on changes in ARG expression [10]. Thus, it is of special significance to identify biomarkers from ARGs that are associated with the prognosis of patients with cancer and to calculate their influence on the prognosis model. Recent reports have shown that prognostic models based on ARGs provide better prediction of clinical outcomes for patients with cancer, such as thyroid cancer and bladder cancer [11, 12]. However, studies on prognostic models based on ARGs in HCC are limited. Thus, our aims in this study were to construct a prognostic model based on ARGs to predict HCC patient survivals.

In this study, we identified the 62 differentially expressed ARGs in HCC based on The Cancer Genome Atlas (TCGA) database. Enrichment analysis of differentially expressed ARGs may help to identify the potential molecular mechanisms in autophagy. In addition, we performed univariate Cox regression analysis to identify 34 prognosis-related ARGs and conducted multivariate Cox regression analysis to select 3 key genes (PRKCD, BIRC5, and ATIC) to construct a prognostic model. Univariate and multivariate Cox regression analyses confirmed that the risk score calculated by the model formula was an independent risk factor for patient survival. Survival analysis and ROC curve analysis demonstrated that the model had good performance in predicting prognosis. Moreover, we confirmed the reliability of this model by analyzing another independent data cohort obtained from the International Cancer Genome Consortium (ICGC) database. In summary, the prognostic model we constructed in this study is not only helpful in identifying the potential mechanism of autophagy but also important for developing an effective prediction tool for HCC patients.

## 2. Materials and Methods

### 2.1. Data Acquisition

The Human Autophagy Database (HADb, http://www.autophagy.lu/index.html) is the first human autophagy-dedicated database. It is a public repository containing information about the human genes involved in autophagy described to date [13]. All ARGs for subsequent analysis were downloaded from this database. The expression data (type: FPKM) and clinical information of HCC were obtained from The Cancer Genome Atlas (TCGA, https://portal.gdc.cancer.gov/) database to identify differentially expressed ARGs and construct the prognostic model, and independent datasets obtained from the International Cancer Genome Consortium (ICGC, https://icgc.org) were used to validate the model. Data were downloaded from the publicly available database; hence, additional ethical approval was not necessary for this study.

### 2.2. Differentially Expressed ARGs and the Enrichment Analysis

The Wilcoxon rank-sum test was used for the identification of differentially expressed ARGs in HCC samples compared with noncancer tissue samples with the criteria of adjusted *p* value < 0.05 and ∣log_2_FC | >1. The Gene Ontology (GO) and Kyoto Encyclopedia of Genes and Genomes (KEGG) pathway analyses were performed using the R clusterProfiler package, and the GO and KEGG results were visualized using the GOplot package. A *p* value < 0.05 was set as the cutoff criterion for both GO and KEGG functional analyses.

### 2.3. Construction and Validation of the Prognostic Model

Univariate Cox regression analysis evaluated the association between the expression of differentially expressed ARGs and patient overall survival (OS). Genes with a *p* value less than 0.05 were considered prognosis-related ARGs and then entered into a stepwise multivariate Cox regression analysis tested by AIC (Akaike Information Criterion, assessing the goodness of fit of a statistical model) to identify the predictive model. Then, a prognostic model was constructed, and the formula of the risk score was as follows: risk score = (the expression of gene_1_ × regression coefficient of gene_1_) + (the expression of gene_2_ × regression coefficient of gene_2_) + ⋯+(the expression of gene_*n*_ × regression coefficient of gene_*n*_).

Based on the risk score, HCC patients were classified into low- or high-risk groups. Survival curves were generated using the Kaplan-Meier method, and two-sided log-rank tests were employed to compare the differences in OS between the low- and high-risk groups. Univariate Cox regression analysis and multivariate Cox regression analysis were conducted to explore whether the risk score could be an independent indicator of OS in the TCGA data cohort of HCC patients. The sensitivity and specificity of the prognostic model to predict the clinical outcome of HCC patients were analyzed by calculating the area under the curve (AUC) of the receiver operating characteristic (ROC) curve. Moreover, an independent data cohort of the ICGC database was used to further confirm the predictive value of the model.

### 2.4. Statistical Analysis

R 3.6.1 (https://www.r-project.org) was utilized for plot and statistical analysis. The Wilcoxon rank-sum test was used to identify differentially expressed ARGs. Univariate Cox regression analysis was performed to estimate the prognosis-related ARGs. Multivariate Cox regression analysis was used to construct the prognostic model. An independent *t*-test was used to analyze the associations between risk score and conventional clinical parameters. ROC analysis was performed to test the sensitivity and specificity of the model. The survival curve was plotted using the survival and survminer packages of R. The forest maps were plotted by the forestplot package of R. The survivalROC package of R was used to generate ROC curves and AUC values to calculate according to ROC curves.

## 3. Results

### 3.1. Differentially Expressed Autophagy-Related Genes between HCC and Normal Tissues

**Figure 1:**
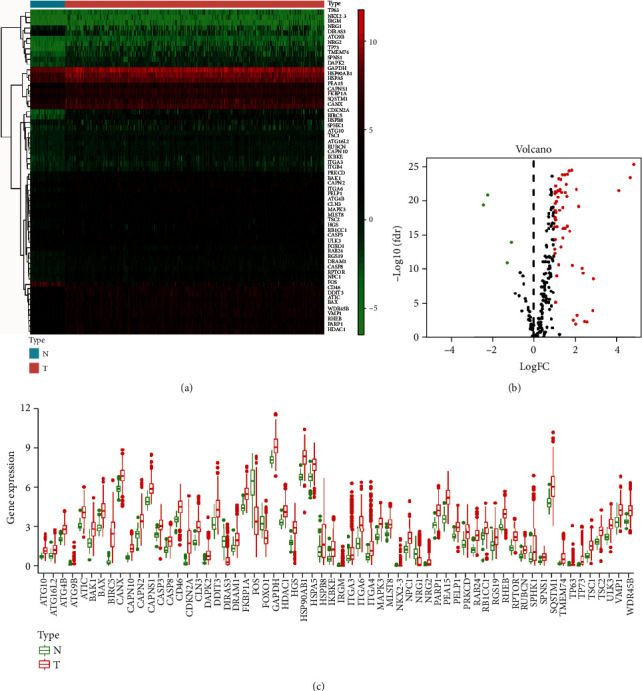
The differentially expressed autophagy-related genes (ARGs) between normal and hepatocellular carcinoma (HCC) tissues. (a) The hierarchical clustering of differentially expressed ARG expression levels. (b) The volcano plot for the differentially expressed ARGs. Red indicates high expression, green indicates low expression, and black indicates no difference between HCC and normal tissue. (c) The boxplot for the expression patterns of 62 significant differentially expressed ARGs in HCC and normal tissue.

To date, HADb datasets include a total of 232 ARGs that have been described as being involved in autophagy. A total of 50 nontumor tissues and 374 tumor tissue samples with mRNA expression profiles and 235 HCC clinical follow-up data points were downloaded from the TCGA database (Table 1). Compared with the noncancer tissue samples, 62 differentially expressed ARGs were identified in the HCC samples (Table 2). The results of hierarchical cluster analysis showed that the HCC samples could be clearly distinguished from the normal tissues based on the differentially expressed ARGs (Figure 1(a)), and the volcano plot showed that there were 4 downregulated genes and 58 upregulated genes among these ARGs (Figure 1(b)). Furthermore, a scatter plot was generated to visualize the expression levels of differentially expressed ARGs between HCC and normal tissue (Figure 1(c)).

### 3.2. Enrichment Analysis of the Differentially Expressed ARGs

**Figure 2:**
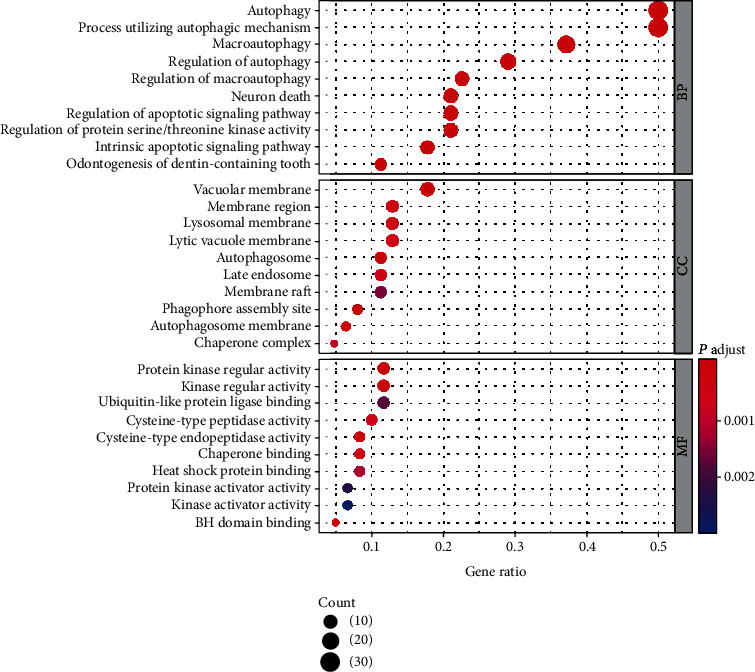
Gene Ontology (GO) enrichment analysis of the differentially expressed autophagy-related genes. The size of each dot represents the count of genes; the color represents the *p*.adjust.

**Figure 3:**
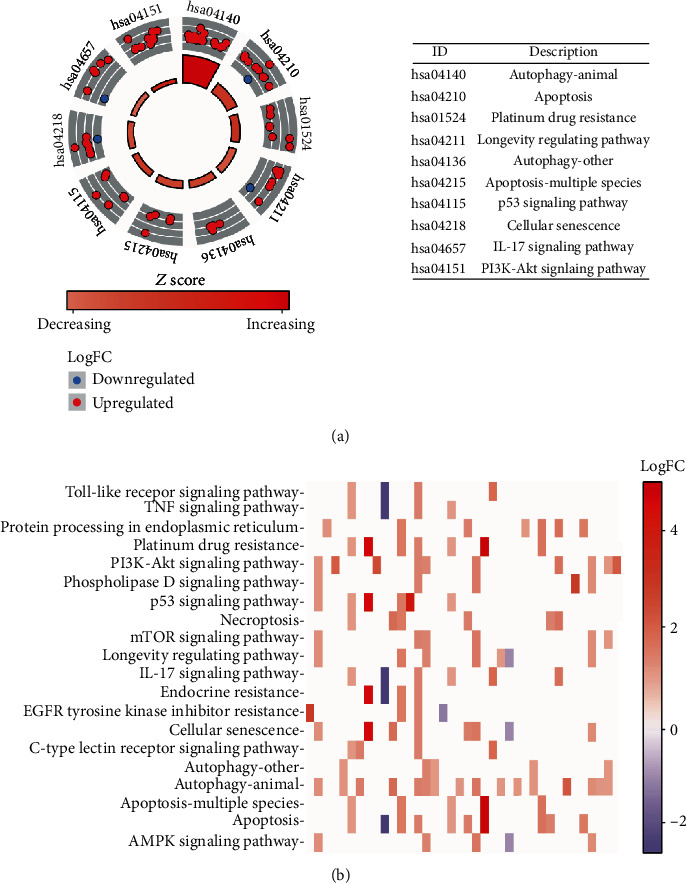
Kyoto Encyclopedia of Genes and Genomes (KEGG) analysis of differentially expressed autophagy-related genes (ARGs). (a) The outer circle shows a scatter plot for each term of the logFC of the assigned gens. Red circles display upregulation, and blue ones display downregulation. (b) The heat map of the relationship between ARGs and enriched pathways. The color of each block depends on the logFC value.

To further investigate the biological functions of the differentially expressed ARGs in HCC, we performed GO term function and KEGG pathway enrichment analyses for these genes (Table 3). For biological processes (BPs), these ARGs participate in autophagy, processes utilizing autophagic mechanisms, macroautophagy, and the regulation of autophagy. In terms of cellular components (CC), the genes were related to autophagosomes, phagophore assembly sites, vacuolar membranes, and autophagosome membranes. Changes in molecular function (MF) were enriched in protein kinase regulator activity, kinase regulator activity, cysteine-type endopeptidase activity, and BH domain binding (Figure 2, Table 3). Furthermore, KEGG pathway analysis showed that these genes mostly participated in autophagy-animal, apoptosis, platinum drug resistance, and longevity regulating pathways (Figures 3(a) and 3(b); Table 4).

### 3.3. Identification of Prognostic-Related ARGs

**Figure 4:**
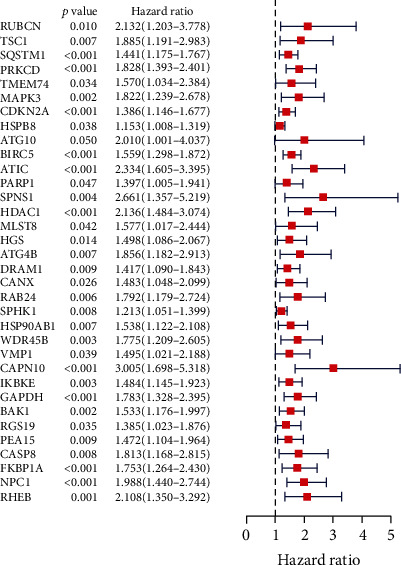
Thirty-four differentially expressed autophagy-related genes with a prognostic value determined by univariate Cox regression.

To identify ARGs associated with the clinical outcomes of patients, univariate Cox regression analysis was applied with the criterion of a *p* value < 0.05. As shown in Figure 4, 34 genes were selected and significantly associated with the overall survival (OS) of HCC patients. The hazard ratio (HR) of each gene was calculated, and all 34 genes had an HR > 1, indicating that all of these genes are risk factors for OS in HCC patients.

### 3.4. Construction and Validation of the ARG-Based Prognostic Model

Based on the prognosis-related ARGs, multivariate Cox regression analysis was performed to construct the ARG-based prognostic model. Three genes, PRKCD, BIRC5, and ATIC, were finally selected to construct the model (Table 5), and the risk score formula of the model was as follows: risk score = (0.3175 × PRKCD expression) + (0.4397 × ATIC expression) + (0.2479 × BIRC5 expression).

**Figure 5:**
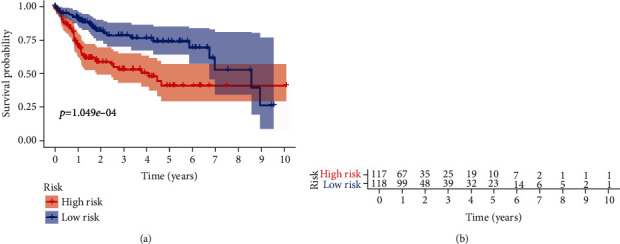
Classification of patients into high- and low-risk groups by median risk score. (a) Kaplan-Meier plot of patients in a low- or high-risk group (*p* = 1.049*e* − 04). (b) The number of patients in different risk groups.

**Figure 6:**
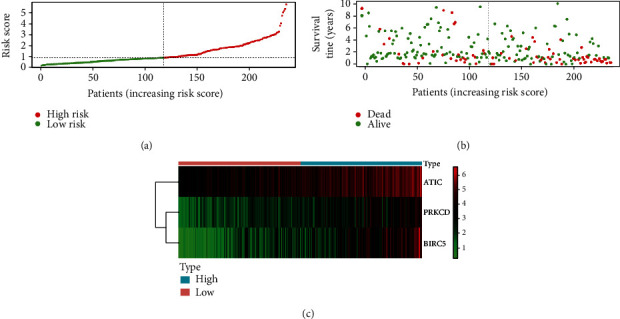
Prognostic risk score of the differentially expressed autophagy-related genes. (a) The risk score of each hepatocellular carcinoma patient. (b) The patient survival based on the risk score. (c) Heat map of the expression of three genes in high- and low-risk groups.

**Figure 7:**
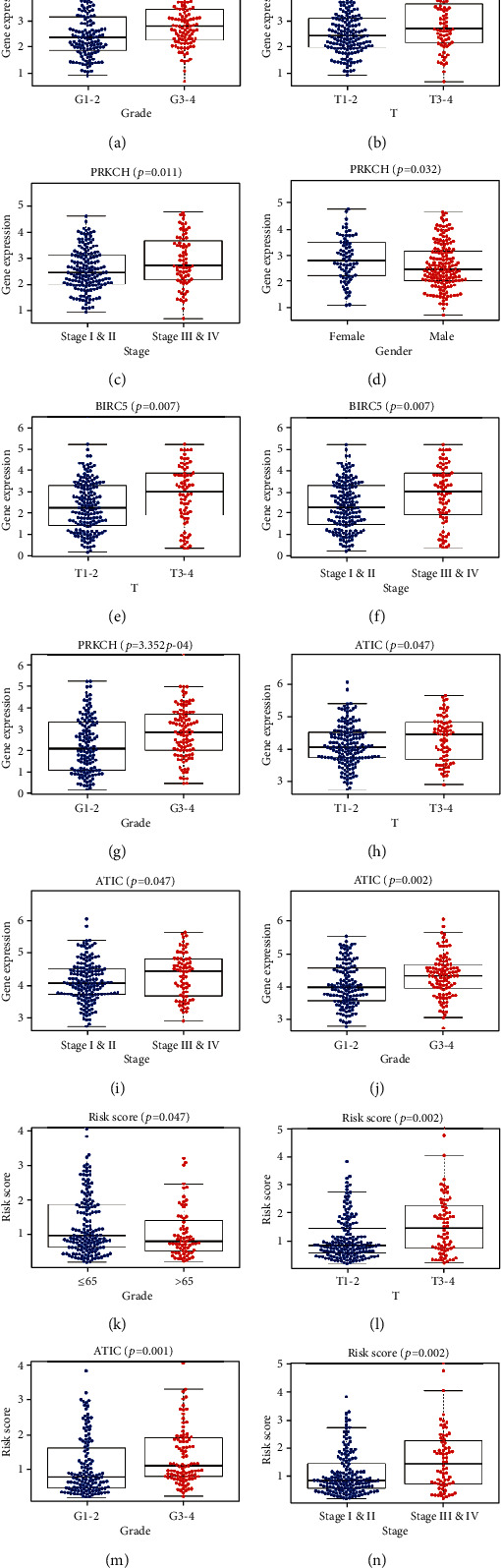
Correlations between expression of three key genes and clinical parameters (the results of *p* < 0.05 are shown).

To assess the performance of the prognostic model in predicting the clinical outcome of HCC patients, we calculated the risk score of each HCC patient and divided patients into high- or low-risk groups using the median risk score as the cutoff value. The survival curve indicated that the high-risk group (*n* = 117) suffered significantly lower 3- and 5-year survival rates than the low-risk group (*n* = 118) (Figure 5). The risk score distribution, survival status of each patient, and heat map of the three gene expression profiles in the TCGA database are shown in Figure 6. The results indicated that survival time decreased and the mortality rate increased as the risk scores increased. We further analyzed the associations between the expression of the three ARGs and clinical parameters in HCC patients. The results of the independent *t*-test are shown in Figure 7. We observed significant correlations between overexpression of PRKCD and advanced histological grade (*p* = 0.001), advanced T stage (*p* = 0.011), advanced pathologic stage (*p* = 0.011), and female sex (*p* = 0.032). High BIRC5 expression was closely related to advanced T stage (*p* = 0.007), advanced pathologic stage (*p* = 0.007), and advanced histological grade (*p* = 3.352*e* − 04). Overexpression of ATIC occurred in the advanced T stage (*p* = 0.047), advanced pathologic stage (*p* = 0.047), and advanced histological grade (*p* = 0.002). In particular, we used the same method to analyze the correlations between the risk score calculated by the model and clinical parameters. As shown in Figures 7(k)–7(n), we found that a high-risk score was significantly related to younger patients (*p* = 0.047), advanced T stage (*p* = 0.002), advanced histological grade (*p* = 0.001), and advanced pathologic stage (*p* = 0.002).

**Figure 8:**
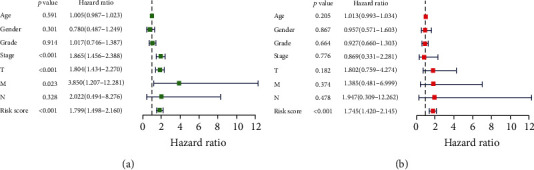
Univariate and multivariate Cox regression analyses of OS in HCC patients. (a) Univariate Cox regression analysis. (b) Multivariate Cox regression analysis. Age: ≤65 vs. >65; gender: male vs. female; grade: G1/G2 vs. G3/G4; stage: I/II vs. III/IV; T stage: T1/T2 vs. T3/T4; M stage: M0 vs. M1; N stage: N0 vs. N1-3; risk score: high-risk score vs. low-risk score (median risk score as the cutoff value).

**Figure 9:**
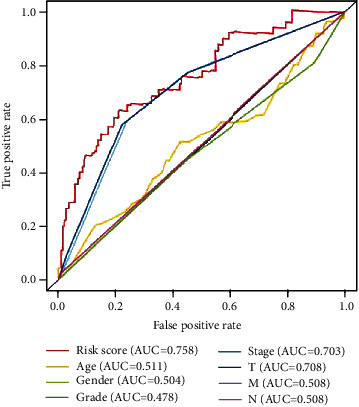
Receiver operating characteristic (ROC) curve analysis for the prognostic value of the prognostic model and other conventional clinical parameters. AUC: area under the curve.

Then, we wanted to determine whether the risk scores or other conventional clinical parameters of HCC patients were independent risk factors for the OS of patients. In Figure 8, univariate Cox analysis showed that advanced pathologic stage, advanced T stage, advanced M stage, and risk score were risk factors for OS. However, after adjusting for clinical parameters, only the risk score remained an independent prognostic indicator for HCC patients in multivariate analyses (HR = 1.745, 95%CI = 1.420‐2.145, *p* < 0.001). Moreover, we plotted the ROC curve to compare the predictive value of the risk score with other clinical parameters, as is shown in Figure 9. The results indicate that the T stage (AUC = 0.708) and pathological stage (AUC = 0.703) have the highest predictive value among the conventional clinical parameters. However, the predictive value of the risk score (AUC = 0.758) was higher than that of the T stage and pathological stage.

### 3.5. Confirmation of the Prognostic Model Using an Additional Data Cohort

**Figure 10:**
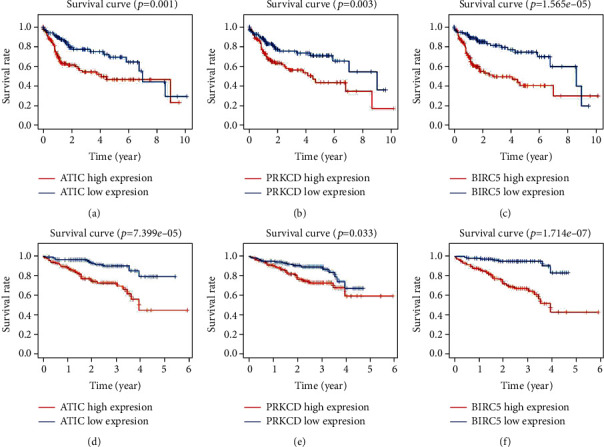
The correlation between the expression level of three genes included in the prognostic model and survival of HCC: (a) ATIC expression level and patient survival (data from TCGA), (b) PRKCD expression level and patient survival (data from TCGA), (c) BIRC5 expression level and patient survival (data from TCGA), (d) ATIC expression level and patient survival (data from ICGC), (e) PRKCD expression level and patient survival (data from ICGC), and (f) BIRC5 expression level and patient survival (data from ICGC).

**Figure 11:**
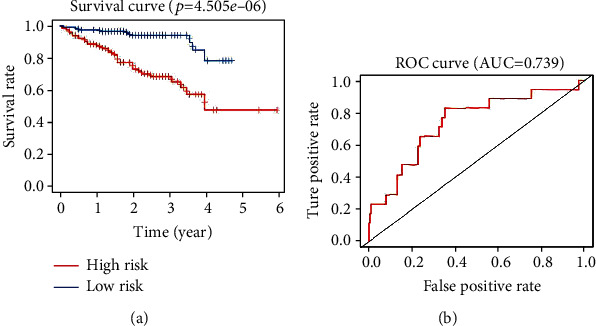
Kaplan-Meier and ROC curves for the ARG prognostic model in the cohort of the ICGC. (a) The differences between the low-risk (*n* = 116) and high-risk (*n* = 116) groups determined by the log-rank test (*p* = 4.505*e* − 06). (b) ROC curve analysis for survival prediction by the prognostic model.

The International Cancer Genome Consortium (ICGC) database contains large-scale cancer genome studies in tumors from 50 different cancer types [14]. To further validate the predictive value of this model, we downloaded the data cohort (Liver Cancer-RIKEN, JP, https://dcc.icgc.org/releases/current/Projects/LIRI-JP), which contains expression profiles and clinical follow-up information from 232 HCC patients. We analyzed the correlation between the expression levels of three genes and the survival of HCC patients in both the TCGA and ICGC databases. Kaplan-Meier analysis results indicated that high expression of the three genes was significantly associated with inferior OS in HCC patients (Figure 10). Similarly, these patients were divided into low- or high-risk groups based on the risk score calculated by the prognostic model constructed based on the TCGA data. The Kaplan-Meier analysis of the two groups was significantly different (*p* = 4.505*e* − 06 < 0.001) and a similar trend was observed in TCGA (Figure 11(a)). In addition, the AUC calculated by the ROC curve was 0.739, which indicated that the model has good performance for predicting HCC patient survival (Figure 11(b)).

## 4. Discussion

The incidence of HCC is characterized by insidiousness, rapid progression, and a low early diagnosis rate. Most patients are already in advanced tumor stages when they receive treatment [15, 16]. Due to the heterogeneity of liver cancer, conventional clinical parameters such as age, sex, grade, and TNM stage often do not accurately predict clinical outcomes [17]. Therefore, there is an urgent need to develop new prognostic features to facilitate the prediction of clinical outcomes in HCC patients. In recent decades, many studies have focused on identifying novel biomarkers to promote the prediction of HCC patient survival [18–20]. Based on the advantages of this type of research, the combination of multiple prognosis-related genes with conventional clinical parameters to construct a prognostic model may have better predictive value for HCC patients.

Autophagy is an intracellular self-digestion process that plays a fundamental role in cell homeostasis, and numerous studies have demonstrated that ARGs play important roles in tumorigenesis [21–23]. Therefore, identifying biomarkers from ARGs may provide new perspectives of the diagnosis or treatment for various cancers. Recently, accumulating reports have shown that using ARGs to build a prognostic model can provide better prediction of clinical outcomes for cancer patients. Lin et al. established a prognostic signature based on three ARGs in thyroid cancer [11]. Wang et al. analyzed TCGA and GEO datasets to construct and validated an autophagy-clinical prognostic model in bladder cancer [12]. Although numerous studies have confirmed the close relationship between the development of HCC and autophagy, a prognostic model based on ARGs in HCC had not been reported previously.

In this study, we used high-throughput expression profile data from TCGA to construct an ARG prognostic model and validated this model using data from TCGA and ICGC. First, we identified 62 ARGs were differentially expressed in tumor tissues. GO analysis and KEGG pathway enrichment analysis were used to explore the potential biological functions of these genes. GO enrichment revealed that these genes were mostly enriched in autophagy, a process utilizing autophagic mechanisms, macroautophagy, and autophagosomes, indicating that the differentially expressed genes mainly affect the progression of liver cancer by affecting autophagy [24, 25]. KEGG pathway enrichment was mainly enriched in autophagy-animal, apoptosis, and platinum drug resistance. The results are consistent with previous studies showing that dysregulated apoptosis and platinum-based resistance are common features of many cancers, including HCC [26–28]. Then, univariate Cox regression analysis identified 34 genes closely related to the survival of HCC patient, and multivariate Cox regression analysis finally selected three key genes (PRKCD, BIRC5, and ATIC) to construct the prognostic model. We divided patients into high- or low-risk groups and found that the high-risk group had a lower 3- or 5-year survival rate. Then, we confirmed that the risk score calculated by the model formula was an independent prognostic indicator after adjusting for other clinical parameters. In addition, ROC curve analysis demonstrated that the risk score has a better predictive value than other clinical parameters. Furthermore, we also observed a similar trend of survival analysis and ROC curve analysis in an independent dataset from the ICGC database which further confirmed the reliability of this prognostic model.

The three ARGs that we have selected to construct the prognostic model have been reported to be involved in the development of cancer in other studies. PRKCD is one of the PKC family members and is a family of serine- and threonine-specific protein kinases that can be activated by calcium and the second messenger diacylglycerol [29]. Previous studies suggested that PRKCD can show completely opposite effects on tumors in different cell types [30]. For example, PRKCD overexpression protected keratinocytes from UV-induced apoptosis and enhanced long-term survival which is a protective mechanism against skin carcinogenesis [31, 32]. LV et al. found that PRKCD can promote tumor progression in pancreatic cancer [33]. In liver cancer, Zhang et al. reported that dihydromyricetin inhibits the migration and invasion of hepatoma cells by reducing MMP-9 expression via a mechanism that is dependent on the upregulation of PRKCD [34]. Nambotin et al. confirmed that Frizzled-7 displays anticancer properties against HCC involving PRKCD activation [35]. In addition, Cai et al. implied that PRKCD is an independent gene involved in the progression of NAFLD to HCC [36]. Combined with our research, we suggest that high expression of PRKCD in HCC may affect tumorigenesis and serve as a biomarker for predicting patient survivals. BIRC5 (also known as survivin) is a member of the inhibitor of the apoptosis (IAP) gene family, which is essential for cell division and can inhibit apoptotic cell death [37, 38]. Ambrosini et al. first described this gene as an oncogene that is prominently expressed in all common human cancers of the lung, colon, pancreas, prostate, and breast [39]. Consistent with the results of previous studies, Chen et al. recently found that increased BIRC5 expressions are associated with histological grade, tumor size, and TNM stage in HCC patients which is consistent with our findings [40]. The protein encoded by ATIC is the last enzyme in the de novo purine biosynthetic pathway [41]. Previous studies have demonstrated that the purine synthesis pathway correlates with cancer cell proliferation [42, 43]. Li et al. recently found that ATIC is an oncogenic gene that promotes survival, proliferation, and migration by targeting AMPK-mTOR-S6K1 signaling in HCC [44]. Although many studies have reported that all three ARGs we selected to construct prognostic models were directly or indirectly involved in various cancers, this is the first study to combine those genes with clinical information to predict the prognosis of HCC.

In summary, we constructed and validated a prognostic model based on three ARGs and this model could be a useful tool to predict the survival of HCC. To our knowledge, the three ARG-related prognostic models have not been reported previously, and the differentially expressed ARGs may provide a new perspective for the study of HCC molecular mechanisms. However, there are some limitations of our research that should be taken into consideration. First, we only focused on the mRNA levels of these genes, and protein levels should be further investigated. Second, the results are exclusively based on the TCGA and ICGC datasets, and additional independent cohorts are necessary to confirm the reliability of this model. Third, the results of our study are descriptive and the potential molecular mechanisms of the three genes warrant additional functional experiments.

## Figures and Tables

**Table 1:** General characteristics of HCC patients included in the present study (data downloaded from the TCGA database).

Parameters	Patients (*N* = 235)	
	Number	%
Age		
≤65 y	164	69.79
>65 y	71	30.21
Gender		
Male	161	68.51
Female	74	31.49
Grade		
G1/2	132	56.17
G3/4	103	43.83
Pathologic stage		
I/II	163	69.36
III/IV	72	30.64
T stage		
T1/2	163	69.36
T3/4	72	30.64
N stage		
N0	231	98.3
N1-3	4	1.7
M stage		
M0	231	98.3
M1	4	1.7

**Table 2:** Characteristics of the differentially expressed autophagy-related genes by using the Wilcoxon rank-sum test (tumor vs. normal).

Gene symbol	logFC	Regulation	*p* value	FDR
BIRC5	4.8146253	Up	2.35*E* − 28	4.82*E* − 26
PEA15	1.83411226	Up	3.57*E* − 27	3.66*E* − 25
RAB24	1.70056392	Up	8.37*E* − 27	4.29*E* − 25
CLN3	1.7043542	Up	7.73*E* − 27	4.29*E* − 25
HSP90AB1	1.63411809	Up	3.53*E* − 26	1.45*E* − 24
MAPK3	1.37850531	Up	5.73*E* − 26	1.68*E* − 24
CAPN10	1.49940395	Up	4.96*E* − 26	1.68*E* − 24
CDKN2A	4.65431982	Up	1.87*E* − 25	4.26*E* − 24
RHEB	1.21520158	Up	2.98*E* − 25	6.10*E* − 24
RPTOR	1.30199062	Up	4.43*E* − 25	8.26*E* − 24
TSC1	1.56728821	Up	2.41*E* − 24	4.11*E* − 23
ATG4B	1.05882354	Up	4.52*E* − 24	7.13*E* − 23
ATIC	1.26886226	Up	1.56*E* − 23	2.18*E* − 22
ITGA6	2.05950447	Up	1.59*E* − 23	2.18*E* − 22
FKBP1A	1.164278	Up	2.89*E* − 23	3.37*E* − 22
TP73	4.10294233	Up	2.96*E* − 23	3.37*E* − 22
ULK3	1.25487092	Up	3.47*E* − 23	3.75*E* − 22
MLST8	1.04550722	Up	4.72*E* − 23	4.84*E* − 22
CAPNS1	1.10213996	Up	5.81*E* − 23	5.42*E* − 22
PARP1	1.34948718	Up	6.82*E* − 23	6.08*E* − 22
BAX	1.52707657	Up	7.52*E* − 23	6.42*E* − 22
ATG10	1.0226174	Up	1.11*E* − 22	9.11*E* − 22
DIRAS3	-2.2142941	Down	2.04*E* − 22	1.49*E* − 21
HGS	1.59409057	Up	3.22*E* − 22	2.28*E* − 21
TSC2	1.1673426	Up	4.51*E* − 22	3.08*E* − 21
CANX	1.10228843	Up	2.00*E* − 21	1.32*E* − 20
WDR45B	1.03610977	Up	4.09*E* − 21	2.62*E* − 20
FOS	-2.4192498	Down	6.68*E* − 21	4.15*E* − 20
DAPK2	2.15868832	Up	1.19*E* − 20	7.20*E* − 20
NPC1	1.40873288	Up	1.77*E* − 20	9.80*E* − 20
GAPDH	1.24841467	Up	5.02*E* − 20	2.64*E* − 19
PELP1	1.02773866	Up	5.19*E* − 20	2.66*E* − 19
HSPA5	1.07141566	Up	1.04*E* − 19	5.18*E* − 19
HDAC1	1.0260015	Up	6.77*E* − 19	2.89*E* − 18
VMP1	1.03638398	Up	1.06*E* − 18	4.45*E* − 18
RUBCN	1.05799379	Up	1.65*E* − 18	6.76*E* − 18
BAK1	1.61591549	Up	1.55*E* − 17	5.67*E* − 17
CD46	1.12531986	Up	1.83*E* − 17	6.60*E* − 17
CASP3	1.01180546	Up	1.98*E* − 17	6.98*E* − 17
DDIT3	1.50655255	Up	3.18*E* − 17	1.10*E* − 16
CAPN2	1.4191918	Up	3.60*E* − 17	1.23*E* − 16
SQSTM1	1.73640938	Up	9.02*E* − 17	2.89*E* − 16
PRKCD	1.41302501	Up	8.94*E* − 16	2.82*E* − 15
CASP8	1.04011948	Up	1.52*E* − 15	4.65*E* − 15
FOXO1	-1.0757149	Down	4.28*E* − 15	1.25*E* − 14
SPNS1	1.31587141	Up	2.15*E* − 14	5.96*E* − 14
RB1CC1	1.01420177	Up	4.73*E* − 14	1.29*E* − 13
ATG16L2	1.02505335	Up	2.00*E* − 13	5.40*E* − 13
NRG1	-1.280118	Down	5.44*E* − 12	1.25*E* − 11
DRAM1	1.2000343	Up	1.11*E* − 11	2.54*E* − 11
IKBKE	1.86880957	Up	1.30*E* − 11	2.93*E* − 11
TMEM74	2.33376628	Up	3.59*E* − 11	7.83*E* − 11
ITGB4	2.37261675	Up	1.85*E* − 10	3.88*E* − 10
RGS19	1.11586497	Up	5.15*E* − 10	1.02*E* − 09
NRG2	2.87978625	Up	1.43*E* − 09	2.68*E* − 09
ATG9B	1.05236942	Up	5.60*E* − 06	8.32*E* − 06
SPHK1	2.84999763	Up	8.75*E* − 05	1.21*E* − 04
ITGA3	2.09689798	Up	4.48*E* − 04	5.89*E* − 04
TP63	1.90806327	Up	2.37*E* − 03	2.93*E* − 03
IRGM	2.45185036	Up	3.98*E* − 03	4.86*E* − 03
NKX2-3	2.56776198	Up	4.50*E* − 03	5.46*E* − 03
HSPB8	2.02223664	Up	9.57*E* − 03	1.13*E* − 02

logFC: log(fold change); FDR: false discovery rate.

**Table 3:** GO analysis of differentially expressed autophagy-related genes.

Category	ID	Term	Count	*p* value
Biological process	GO:0006914	Autophagy	31	8.54*E* − 33
Biological process	GO:0061919	Process utilizing autophagic mechanism	31	8.54*E* − 33
Biological process	GO:0016236	Macroautophagy	23	2.05*E* − 26
Biological process	GO:0010506	Regulation of autophagy	18	1.38*E* − 17
Biological process	GO:0016241	Regulation of macroautophagy	14	3.91*E* − 16
Biological process	GO:0070997	Neuron death	13	1.53*E* − 10
Biological process	GO:2001233	Regulation of apoptotic signaling pathway	13	7.96*E* − 10
Biological process	GO:0097193	Intrinsic apoptotic signaling pathway	11	2.51*E* − 09
Biological process	GO:0071900	Regulation of protein serine/threonine kinase activity	13	7.65*E* − 09
Biological process	GO:0042475	Odontogenesis of dentin-containing tooth	7	1.68*E* − 08
Cellular component	GO:0005776	Autophagosome	7	1.26*E* − 08
Cellular component	GO:0000407	Phagophore assembly site	5	1.38*E* − 08
Cellular component	GO:0005774	Vacuolar membrane	11	4.53*E* − 08
Cellular component	GO:0000421	Autophagosome membrane	4	2.83*E* − 06
Cellular component	GO:0098589	Membrane region	8	1.05*E* − 05
Cellular component	GO:0005770	Late endosome	7	1.43*E* − 05
Cellular component	GO:0005765	Lysosomal membrane	8	1.74*E* − 05
Cellular component	GO:0098852	Lytic vacuole membrane	8	1.74*E* − 05
Cellular component	GO:0101031	Chaperone complex	3	3.25*E* − 05
Cellular component	GO:0045121	Membrane raft	7	7.15*E* − 05
Molecular function	GO:0019887	Protein kinase regulator activity	7	4.93*E* − 07
Molecular function	GO:0019207	Kinase regulator activity	7	1.14*E* − 06
Molecular function	GO:0004197	Cysteine-type endopeptidase activity	5	1.55*E* − 06
Molecular function	GO:0051400	BH domain binding	3	4.96*E* − 06
Molecular function	GO:0008234	Cysteine-type peptidase activity	6	1.35*E* − 05
Molecular function	GO:0051087	Chaperone binding	5	1.72*E* − 05
Molecular function	GO:0031072	Heat shock protein binding	5	3.93*E* − 05
Molecular function	GO:0044389	Ubiquitin-like protein ligase binding	7	6.46*E* − 05
Molecular function	GO:0030295	Protein kinase activator activity	4	9.71*E* − 05
Molecular function	GO:0019209	Kinase activator activity	4	0.000135

**Table 4:** KEGG analysis of differentially expressed autophagy-related genes.

ID	Term	Count	*p* value
hsa04140	Autophagy-animal	16	2.50*E* − 16
hsa04210	Apoptosis	10	2.02*E* − 08
hsa01524	Platinum drug resistance	7	5.52*E* − 07
hsa04211	Longevity regulating pathway	7	2.15*E* − 06
hsa04136	Autophagy-other	5	2.24*E* − 06
hsa04215	Apoptosis-multiple species	5	2.24*E* − 06
hsa04115	p53 signaling pathway	6	8.78*E* − 06
hsa04218	Cellular senescence	8	1.14*E* − 05
hsa04657	IL-17 signaling pathway	6	3.84*E* − 05
hsa04151	PI3K-Akt signaling pathway	10	1.19*E* − 04

**Table 5:** The three ARG-based prognostic models identified by multivariate Cox regression analysis.

Gene symbol	Coef	HR	HR.95L	HR.95H	*p* value
PRKCD	0.3175	1.3737	1.0055	1.8767	0.0461
BIRC5	0.2479	1.2814	1.0194	1.6108	0.0337
ATIC	0.4397	1.5523	1.0038	2.4006	0.0481

## Data Availability

The data were provided by the Human Autophagy Database (HADb, http://www.autophagy.lu/index.html), The Cancer Genome Atlas (TCGA, https://portal.gdc.cancer.gov/), and the International Cancer Genome Consortium (ICGC, https://icgc.org).
